# Preclinical Pharmacokinetics, Tissue Distribution, and Plasma Protein Binding of Sodium (±)-5-Bromo-2-(α-Hydroxypentyl) Benzoate (BZP), an Innovative Potent Anti-ischemic Stroke Agent

**DOI:** 10.3389/fphar.2016.00255

**Published:** 2016-08-18

**Authors:** Xin Tian, Hong-Meng Li, Jing-Yao Wei, Bing-Jie Liu, Yu-Hai Zhang, Gao-Ju Wang, Jun-Biao Chang, Hai-Ling Qiao

**Affiliations:** ^1^Institute of Clinical Pharmacology, Zhengzhou UniversityHenan, China; ^2^Department of Pharmacy, The First Affiliated Hospital of Zhengzhou UniversityHenan, China; ^3^College of Chemistry and Molecular Engineering, Zhengzhou UniversityZhengzhou, China

**Keywords:** BZP, metabolite, Br-NBP, pharmacokinetics, tissue distribution, plasma protein binding

## Abstract

Sodium (±)-5-bromo-2-(α-hydroxypentyl) benzoate (BZP) is a potential cardiovascular drug and exerts potent neuroprotective effect against transient and long-term ischemic stroke in rats. BZP could convert into 3-butyl-6-bromo-1(3*H*)-isobenzofuranone (Br-NBP) *in vitro* and *in vivo*. However, the pharmacokinetic profiles of BZP and Br-NBP still have not been evaluated. For the purpose of investigating the pharmacokinetic profiles, tissue distribution, and plasma protein binding of BZP and Br-NBP, a rapid, sensitive, and specific method based on liquid chromatography coupled to mass spectrometry (LC-MS/MS) has been developed for determination of BZP and Br-NBP in biological samples. The results indicated that BZP and Br-NBP showed a short elimination half-life, and pharmacokinetic profile in rats (3, 6, and 12 mg/kg; *i.v.*) and beagle dogs (1, 2, and 4 mg/kg; *i.v.gtt*) were obtained after single dosing of BZP. After multiple dosing of BZP, there was no significant accumulation of BZP and Br-NBP in the plasma of rats and beagle dogs. Following *i.v.* single dose (6 mg/kg) of BZP to rats, BZP and Br-NBP were distributed rapidly into all tissues examined, with the highest concentrations of BZP and Br-NBP in lung and kidney, respectively. The brain distribution of Br-NBP in middle cerebral artery occlusion (MCAO) rats was more than in normal rats (*P* < 0.05). The plasma protein binding degree of BZP at three concentrations (8000, 20,000, and 80,000 ng/mL) from rat, beagle dog, and human plasma were 98.1–98.7, 88.9–92.7, and 74.8–83.7% respectively. In conclusion, both BZP and Br-NBP showed short half-life, good dose-linear pharmacokinetic profile, wide tissue distribution, and different degree protein binding to various species plasma. This was the first preclinical pharmacokinetic investigation of BZP and Br-NBP in both rats and beagle dogs, which provided vital guidance for further preclinical research and the subsequent clinical trials.

## Introduction

Ischemic stroke accounts for 75–85% of all the strokes occurring annually in China and approximately 5.7 million people are estimated to die of acute ischemic stroke per year worldwide (Bravata et al., 2007; Broussalis et al., 2012). Ischemic stroke is due to the occlusion of an artery in the cerebral circulation by atherosclerotic plaque, thrombus or embolus. The course of the disease is difficult to predict, but it generally involves progressive deterioration (Beal, 2010; Holodinsky et al., 2016). The prevalence of cerebral ischemic stroke is particularly high and the survivors have more or less neurological function deficits, which give rise to a large burden on society and patients' families (Feigin et al., 2015; Truelsen et al., 2015; Wu et al., 2016).

1-3-n-butylphthalide (NBP), first isolated from the seeds of celery, was a potent, and widely used drug for the treatment of ischemic stroke in clinic, and it was approved in form of soft capsule and infusion drip by the China Food and Drug Administration (CFDA) in 2004 (Zhang et al., 2012; Yang et al., 2015; Zhao et al., 2016). NBP displays neuroprotective effects, improves cognitive impairment, and prevents neuronal cell death after focal cerebral ischemia in mice via the c-Jun *N*-terminal kinase pathway (Peng et al., 2008, 2010; Li et al., 2010). NBP underwent extensive metabolism after oral administration, and renal excretion was the primary elimination pathway. However, toxic reactions may arise due to high plasma exposures of the metabolites than that of NBP itself (Diao et al., 2013, 2014). About 28% of the patients had adverse events (AE) such as cerebral hemorrhage, erythra, pneumonia, and liver dysfunction, which may interrupt clinical uses of NBP. In addition, the bioavailability being as low as 15% limits the application of NBP capsule in acute ischemic stroke patients (Cui et al., 2013).

**Figure 1:**
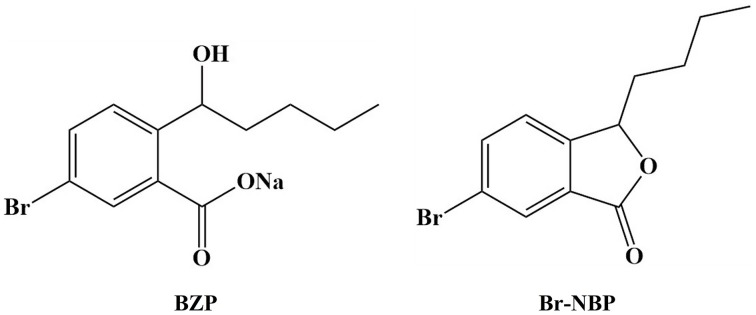
**Chemical structures of BZP and Br-NBP**.

A larger number of NBP derivatives have been explored and biological evaluated in order to improve the pharmacological activities of NBP (Wang et al., 2013; Sheng et al., 2015; Yin et al., 2016). Our previous study showed that 3-butyl-6-bromo-1(3*H*)-isobenzofuranone (Br-NBP; Figure 1) could attenuate hydrogen peroxide-induced damage and platelet aggregation (Gao et al., 2010; Wang et al., 2010; Ma et al., 2012). As the drug was aimed to apply at the acute phase in ischemic stroke, the ideal preparation should be injection formulation. However, the poor water-solubility of Br-NBP limited our intention, so we developed sodium (±)-5-bromo-2-(α-hydroxypentyl) benzoate (BZP) (Figure 1), with improved solubility, to prepare its injection formulation. BZP could convert to Br-NBP in major tissues *in vitro* and *in vivo* (Tian et al., 2016).

BZP is an innovative drug that shows potent anti-ischemic stroke outcomes and was approved for clinical trials by the CFDA (the approval number 2016L01072). Pharmacodynamic study showed that BZP could protect neurological function and decrease infarct volume after middle cerebral artery occlusion (MCAO) with a dose-dependent manner in rats via NF-κB pathway and mitochondrial apoptotic pathway. In addition, BZP markedly improve neurological deficit and exert neuroprotective effects against permanent focal cerebral ischemia in rats. Moreover, BZP could inhibit platelet aggregation and improve dyskinesia and prevent ischemic stroke in salt-sensitive rats (data not shown).

Due to the potent therapeutic effect against ischemic stroke, it is worthwhile to systematically investigate the preclinical pharmacokinetics of BZP and its bioactive metabolite Br-NBP. In light of these concerns, the primary aims of current work were to (1) investigate the non-clinical pharmacokinetic properties of BZP and Br-NBP in rats and beagle dogs. (2) Evaluate the tissue distribution of BZP and Br-NBP in rats. (3) Characterize protein binding rates of BZP and Br-NBP in various species plasma.

## Materials and methods

### Chemical and reagents

BZP bulk drug (purity 99.4%), Br-NBP (purity 99.8%), and PHPB (internal standard, IS, purity 98.5%) were acquired from College of Chemistry and Molecular Engineering, Zhengzhou University (Zhengzhou, China). BZP aseptic powder needle for injection containing 50 mg bulk drug with a total weight of 134 mg per bottle were provided by Beijing Yiscon Technology Co., Ltd. (Beijing, China). Potassium 2-(1-hydroxypentyl)-benzoate, PHPB (internal standard, IS, purity 98.5%) was synthesized at College of Chemistry and Molecular Engineering, Zhengzhou University. NBP (internal standard, IS, purity 99.5%) was purchased from CSPC NBP pharmaceutical Co., Ltd. (Shijiazhuang, China). Methanol (HPLC grade) was obtained from Fisher USA. Ammonium acetate (analytically pure) was purchased from Sinopharm Chemical Reagent Co., Ltd. (Shanghai, China). Purified water from a Milli-Q system (Millipore, Bedford, MA, USA) was used throughout. All other chemicals were of analytical grade and used without further purifications.

### Experimental animals

Sprague Dawley (SD) rats, weighing 200–240 g, were obtained from Beijing Vital River Laboratory Animal Technology Co., Ltd. (Beijing, China). Beagle dogs, weighing 6.5–7.7 kg, were purchased from Beijing Marshall Biotechnology Co., Ltd. (Beijing, China).

Animals were housed under ideal laboratory conditions (temperature 23–25°C, 12 h light/12 h darkness cycle, 45–55% relative humidity) and maintained on standard pellet diet and water *libitum* throughout the experimental period. The animals were fasted overnight with free access to water for at least 12 h before administration. Prior to pharmacokinetic investigations of BZP, beagle dogs received a series of examinations to ensure animal health.

This study was performed according to the Guide for the Care and Use of Laboratory Animals. All experimental procedures reported herein were reviewed and approved by the Zhengzhou University Animal Care and Use Committee.

### Pharmacokinetic studies

#### *In vivo* PK profile of rats

The BZP solution for injection was prepared by dissolving 251.5 mg BZP in 50 mL of 0.9% saline. The BZP solution for infusion was prepared by dissolving 50 mg BZP aseptic powder needle for injection in 5 mL of 0.9% saline.

Thirty rats were randomly divided into three groups (*n* = 10, each group, half male and half female). For single pharmacokinetic studies, the rats were treated with low (3 mg/kg), middle (6 mg/kg), and high (12 mg/kg) doses of BZP via tail vein. Blood samples (200 μL) for pharmacokinetic analyses were collected pre-dose and at 1, 5, 10, 20, 40, 70, 110, 170, 240, and 360 min post-BZP dose by orbital bleeding via heparinized capillary tubes.

For multiple pharmacokinetic studies, the same rats of middle dose group received multiple doses of 6 mg/kg/day for 7 consecutive days after the single-dose study. The blood samples were collected immediately prior to dosage at days 5–6 and pre-dose and at 1, 5, 10, 20, 40, 70, 110, 170, 240, and 360 min post-BZP dose at the last day. Plasma samples were harvested by centrifuging the blood at 4500 rpm for 10 min at 4°C and stored at −80°C until analysis.

#### *In vivo* PK profile of beagle dogs

Twenty-four Beagle dogs were randomly divided into three groups (*n* = 8, each group, half male and half female). For single pharmacokinetic studies, the rats were treated with low (1 mg/kg), middle (2 mg/kg), and high (4 mg/kg) doses of BZP solution for infusion via forelimb vein. The volume for intravenous drip was 50 mL and the time was 20 min. Blood samples (250 μL) for pharmacokinetic analyses were collected at 2, 7, and 13 min during the intravenous drip and at 0, 5, 10, 20, 30, 40, 60, 80, 110, 140, and 180 min post-BZP dose by hind limb vein bleeding via heparinized capillary tubes.

For multiple pharmacokinetic studies, the same beagle dogs of the middle dose group received multiple doses of 2 mg/kg/day for 7 consecutive days after the single-dose study. The beagle dogs were free access to water and food at 2 h after intravenous drip. The blood samples were collected immediately prior to dosage at days 5–6 and collected at 2, 7, and 13 min during the intravenous drip and at 0, 5, 10, 20, 30, 40, 60, 80, 110, 140, and 180 min post-BZP dose at the last day. Sample preparation was consist with those described previously.

### Tissue distribution

#### Tissue distribution of rats

Thirty rats were randomly divided into five groups (*n* = 6, each group, half male and half female), which were treated with a single intravenous administration of BZP (6 mg/kg).Tissues (lung, small intestine, spleen, ovary, large intestine, kidney, liver, heart, stomach, fat, testicles, brain, and muscle) were promptly harvested at 1, 10, 20, 50, and 110 min (six rats/time point) and thoroughly in ice cold saline to eliminate blood and other content and blotted dry with filter paper. An accurately weighted amount of each tissue samples were homogenized in methanol-water (50:50, v/v) solution (5 times the tissue weight, w/v) and stored at −80°C until analysis.

#### Tissue distribution of brain in MCAO rats and normal rats

Twelve male rats were randomly divided into two groups (*n* = 6, each group). One group was anesthetized, and the common carotid artery was separated from the vagus nerve, and blunt dissection was performed to isolate the internal carotid artery (ICA), the external carotid artery (ECA), and the middle cerebral artery (MCA). A 30-mm monofilament was introduced into the ECA, fed distally into the ICA, and advanced approximately 18 mm through the Circle of Willis to the origin of the MCA. The monofilament was then secured into the vessel to achieve occlusion. After 2 h of MCA occlusion, reperfusion was achieved by withdrawal of the suture from external carotid artery. After closing the skin incision, rats were allowed to recover from anesthesia, returned to their cages, and allowed to move and eat freely. The other group did not be disposed as control group.

Then two groups were intravenous received a single dose of 6 mg/kg BZP. The brains were harvested at 1 and 10 min and the samples were disposed as the same with those described in tissue distribution.

### Plasma protein binding of BZP *in vitro*

Ultrafiltration method was adopted in this study, which was widely used to determine the plasma protein binding (Wang and Williams, 2013; Amini et al., 2014; Li et al., 2015). *In vitro* experiment, binding of BZP to protein in fresh plasma from pooled SD rat, beagle dog, and human plasma were measured separately. Plasma samples containing BZP were prepared *in vitro*. The plasma samples were spiked to give final BZP concentrations of 8000, 20,000, and 80,000 ng/mL. The samples were incubated for 30 min at 37°C and 400 μL aliquots were placed into an ultrafiltration device. The samples were centrifuged at 5000 g for 20 min. Concentrations of BZP in the filtrate were determined by the LC-MS/MS. The stability of BZP and Br-NBP in solution was investigated and met the requirements of this study.

### Sample preparation

Each sample was thawed and vortexed. For every 50 μL plasma (or tissue homogenates) samples, 50 μL mixed IS (consisted of PHPB and NBP) and 150 μL methanol were added. The mixture was mixed for 1 min and centrifuged for 10 min at 13,000 rpm. 200 μL supernatant was centrifuged at 13,000 rpm for 5 min again. After centrifugation, supernatant was transferred to a fresh vial and 10 μL was injected into chromatographic system for analysis.

### Bioanalytical methods

Plasma and tissue homogenates concentrations of BZP and Br-NBP were quantified by a sensitive and specific LC-MS/MS method. The assays were validated according to US Food and Drug Administration (USFDA) guidelines on bioanalytical method validation (US Department of Health Human Services et al., 2001).

LC-MS/MS system consisting of a Thermo Fisher ACCELA LC system connected with a Thermo Fisher TSQ QUANTUM ULTRA triple-quadrupole mass spectrometer with an electrospray ionization (ESI) interface in positive and negative-ion mode. Chromatographic separation was achieved on a Hypersil GOLD C_18_ colume (5 μm, 100 × 2.1 mm) maintained at 30°C at a flow rate of 0.2 mL/min. Elution was performed by a gradient mobile phase consisting of 5 mM ammonium acetate (A) and methanol (B). The gradient started at 55% B for 2 min, increasing linearly to 90% B within 2 min, then maintained at 90% B for another 1 min, afterwards level of B was decreased back to 55% within 0.1 min, then the column was equilibrated till 8 min for next sample injection. The injection volume was 10 μL in partial loop mode. The parameter settings were as follows: spray voltage of positive-ion, 3500 V; spray voltage of negative-ion, 2500 V; capillary temperature, 350°C; sheath gas (nitrogen), 30 arbitrary units; auxiliary gas (nitrogen), eight arbitrary units; vaporizer temperature, room temperature. Detection and quantification of the compounds were performed in the multiple reaction monitoring (MRM) mode with the transitions of m/z 285.00 → 85.00 for BZP and m/z 207.00 → 85.00 for PHPB (IS of BZP), m/z 269.00 → 144.00 for Br-NBP and m/z 191.00 → 145.00 for NBP (IS of Br-NBP), respectively. BZP and PHPB were measured in negative-ion and a positive ion mode was used for quantification of Br-NBP and NBP. Thermo Fisher LCQUAN quantitative software was used for data analysis.

Base on the analytical conditions mentioned above, BZP, PHPB (IS of BZP), Br-NBP, and NBP (IS of Br-NBP) were well separated and there was no interference from plasma and tissue homogenates. The assays for plasma and tissue homogenate samples were linear over a range of concentrations from 5 to 10,000 ng/mL (*R*^2^ > 0.99). The lower limit of quantification (LLOQ) value of BZP and Br-NBP both are 5 ng/mL. The lower limit of detection (LLOD) value of BZP and Br-NBP both are 1 ng/mL. Separate sets of analytical quality control samples (for concentrations of 10, 250, and 5000 ng/mL) were used during plasma and tissue homogenate sample and (for concentrations of 10, 50, and 250 ng/mL) were used to assess assay precision and accuracy. The intra- and inter-day precision was lower than 15% in terms of relative standard deviation (RSD). The stability results showed plasma and tissue homogenate samples were stable in three freeze/thaw cycles at −20 and −80°C, 1 month at −20 and −80°C, 24 h at room temperature and 24 h before injection.

### Pharmacokinetic calculation and data analysis

The concentration vs. time data was assessed via non-compartmental analysis using the DAS 2.0 package (version 2.0 pharmacokinetic software; Chinese pharmaco-logical Assn., Beijing, China). The *C*_max_, AUC, and CLz data between different groups were compared using One-way analysis of variance (ANOVA) followed by Tukey's *post-hoc* test. The results are expressed as the mean ± SD. A value of *P* < 0.05 was considered to be statistically significant. All statistical analyses were performed with SPSS 17.0 for windows.

## Results

### Plasma pharmacokinetic study

#### *In vivo* PK in rats

##### Single dose of BZP in rats

**Figure 2:**
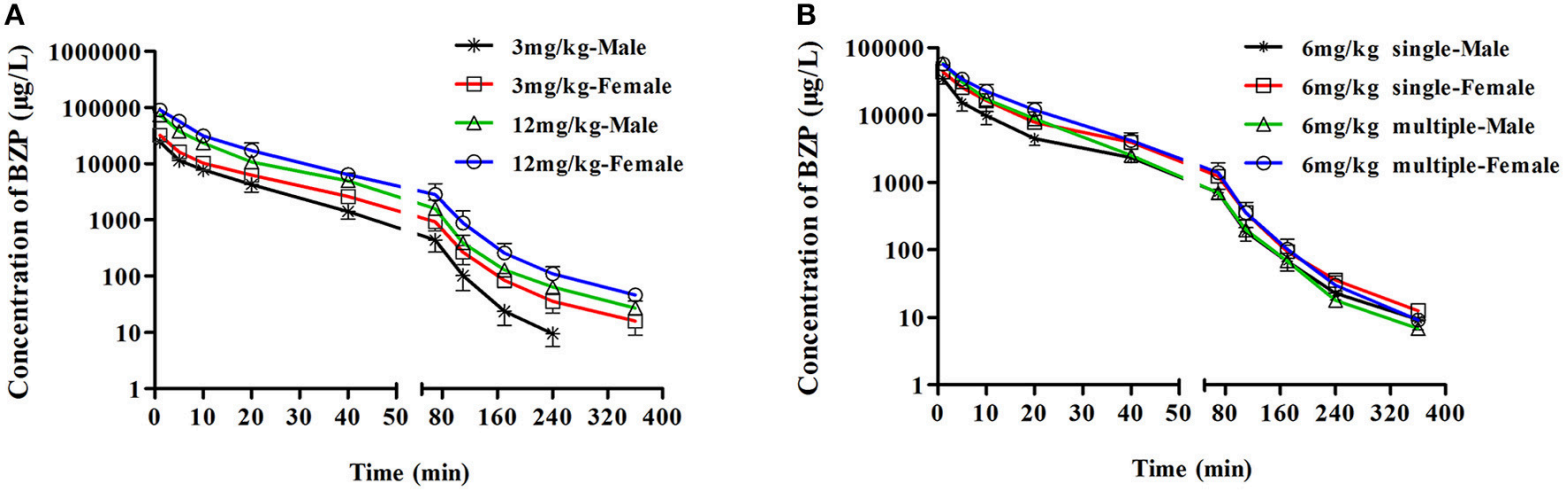
**The mean plasma concentration-time profiles of BZP in rats**. Rats received intravenous administration of BZP **(A)** at a single dose of 3 or 12 mg/kg (*n* = 10); **(B)** at a dose of 6 mg/kg once for 1 day and the same dose for 7 days (*n* = 10). The results are the mean ± SD of the indicated number of rats.

The mean plasma concentration-time profiles of BZP after administration of BZP (3, 6, and 12 mg/kg, *i.v.*) were illustrated in Figure 2. The pharmacokinetic parameters of BZP are presented in Table 1. The mean *C*_max_ and AUC_0–t_ values of BZP were linearly related to the dose in the range of 3–12 mg/kg. The coefficient of association was 0.9792 for dose to AUC_0–t_ in rats (*y* = 88.78x + 71.52; *y* refers to AUC_0–t_, x refers to dose). The plasma concentration declined rapidly following the intravenous dosing with a short t_1/2_. In addition, for three doses, the AUC_0–t_ and *C*_max_ values of BZP for female rats were 1–2 times higher than those of the male rats (*P* < 0.05). As for other major pharmacokinetic parameters such as t_1/2z_, CL_z_, and V_z_, a significant difference was observed between the low dose group (3 mg/kg) and middle dose group (6 mg/kg), while no difference was found between the middle dose group (6 mg/kg) and high dose group (12 mg/kg).

**Table 1:** **The pharmacokinetic parameters of BZP in rats after single and multiple intravenous administration of BZP (***n*** = 10)**.

**PK parameters**	**BZP (3.0 mg/kg) single**		**BZP (6.0 mg/kg) single**		**BZP (12.0 mg/kg) single**		**BZP (6.0 mg/kg) multiple**	
	**Male**	**Female**	**Male**	**Female**	**Male**	**Female**	**Male**	**Female**
AUC_0–t_ (mg·h/L)	302.51 ± 51.23	462.81 ± 50.08^*^	414.13 ± 60.01	659.73 ± 117.10^*^	953.78 ± 142.86	1159.28 ± 337.39^*^	685.15 ± 85.79*^##^*	856.73 ± 179.84^*^^##^
AUC_0–∞_ (mg·h/L)	302.58 ± 51.25	463.18 ± 49.95^*^	414.35 ± 60.04	660.59 ± 117.03^*^	956.86 ± 140.82	1162.45 ± 336.76^*^	685.48 ± 85.82*^##^*	857.00 ± 179.97^*^^##^
MRT_0–t_ (min)	17.25 ± 2.84	24.83 ± 2.11^*^	22.14 ± 2.72	22.99 ± 4.04^*^	21.16 ± 2.20	24.09 ± 2.95^*^	16.66 ± 0.86	20.51 ± 3.20^*^
MRT_0–∞_ (min)	17.31 ± 2.86	25.16 ± 2.22^*^	22.35 ± 2.65	23.60 ± 3.57^*^	22.88 ± 1.84	25.27 ± 2.46^*^	16.86 ± 0.83	20.63 ± 3.27^*^
t_1/2z_ (min)	21.84 ± 1.01	41.23 ± 8.66^*^	42.59 ± 10.03^Δ^	62.62 ± 24.61^*^^Δ^	81.99 ± 36.27^ΔΔ^	67.18 ± 24.01^*^^ΔΔ^	45.12 ± 11.34	38.90 ± 6.54^*^
CL_z_ (L/min·kg)	0.010 ± 0.002	0.007 ± 0.001	0.015 ± 0.002^Δ^	0.009 ± 0.002^Δ^	0.013 ± 0.002^Δ^	0.009 ± 0.003^Δ^	0.009 ± 0.001*^##^*	0.007 ± 0.002*^##^*
V_z_ (L/kg)	0.32 ± 0.05	0.39 ± 0.11	0.90 ± 0.25^ΔΔ^	0.87 ± 0.47^ΔΔ^	1.57 ± 0.82^ΔΔ^	0.94 ± 0.49^*^^ΔΔ^	0.57 ± 0.14*^##^*	0.41 ± 0.10^*^^##^
*C*_max_	25,527 ± 2946	32,123 ± 2644^*^	34,641 ± 5778	43,810 ± 5894^*^	74,785 ± 6822	90,434 ± 11,408^*^	58,309 ± 4158*^##^*	56,888 ± 13,774^*^^##^

* P < 0.05, compared with male group;

Δ *P < 0.05*,

ΔΔ P < 0.01, compared with the group (3.0 mg/kg);

## P < 0.01, compared with the single group (6.0 mg/kg).

##### Multiple dose of BZP in rats

The mean plasma concentration-time profiles of BZP after BZP treatment (6 mg/kg, once daily for 7 days, *i.v.*) were shown in Figure 2. The pharmacokinetic parameters of BZP are presented in Table 1. The *C*_min_ values of BZP are under the LLOQ (5 ng/mL) at 5–7 day. The *C*_max_ and AUC_0–t_ values of BZP were no significant differences in female and male rats after 7 consecutive day's administration. Compared with the parameters after single dose of BZP (6 mg/kg, *i.v.*), the *C*_max_ and AUC_0–t_ values were much higher (*P* < 0.01), the CL_z_ and V_z_ values were much lower (*P* < 0.01), and the t_1/2_ values were no significant differences after 7 consecutive day's administration.

##### PK profile of Br-NBP after single dose of BZP in rats

**Figure 3:**
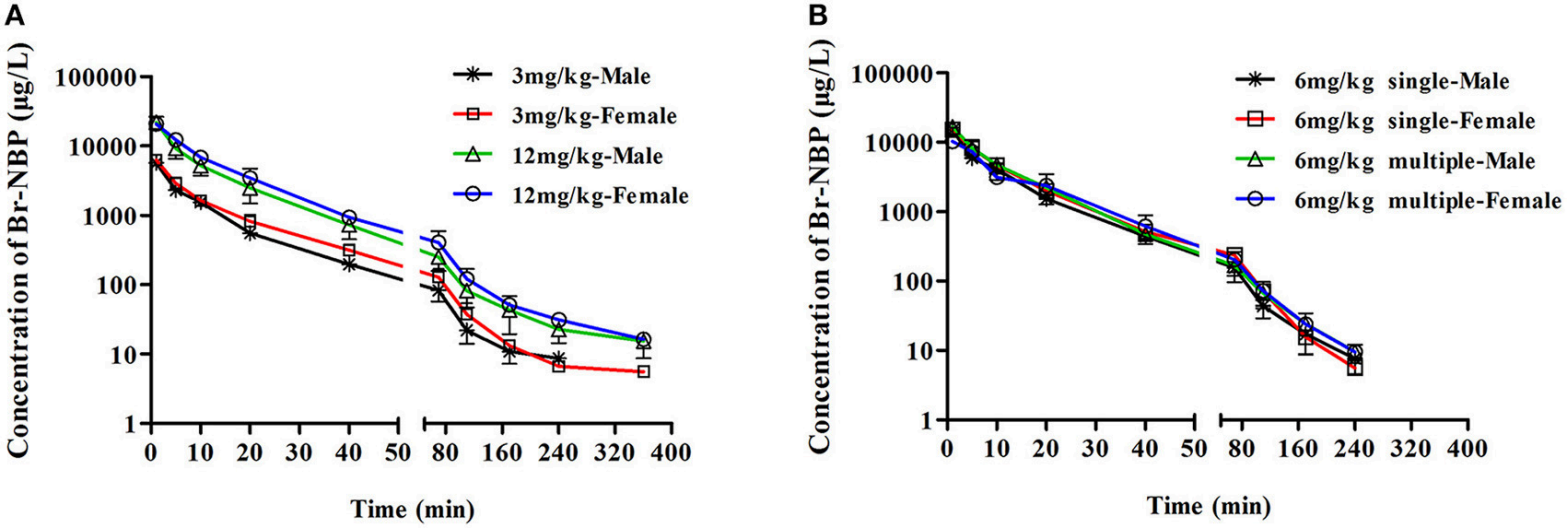
**The mean plasma concentration time profiles of Br-NBP in rats**. Rats received intravenous administration of BZP **(A)** at a single dose of 3 or 12 mg/kg (*n* = 10); **(B)** at a dose of 6 mg/kg once for 1 day and the same dose for 7 days (*n* = 10). The results are the mean ± SD of the indicated number of rats.

As presented in Figure 3, Table 2, the mean *C*_max_ and AUC_0–t_ values of Br-NBP were linearly related to the dose in the range of 3–12 mg/kg. The coefficient of association was 0.9386 for dose to AUC_0–t_ in rats (*y* = 21.83x). In addition, the *C*_max_ values of Br-NBP were no significant differences between female and male rats in the dose of 3–12 mg/kg. The AUC_0–t_ values of Br-NBP were no significant differences in female and male rats in 6 and 12 mg/kg. But for 3 mg/kg, the AUC_0–t_ values of Br-NBP were higher in female rats than in male rats (*P* < 0.05). Similar to BZP, other major pharmacokinetic parameters (t_1/2z_, CLz, and Vz) for Br-NBP were significantly different between low dose group (3 mg/kg) and middle dose group (6 mg/kg), while were same between middle dose group (6 mg/kg) and high dose group (12 mg/kg).

**Table 2:** **The pharmacokinetic parameters of Br-NBP in rats after single and multiple intravenous administration of BZP (***n*** = 10)**.

**PK parameters**	**BZP (3.0 mg/kg) single**		**BZP (6.0 mg/kg) single**		**BZP (12.0 mg/kg) single**		**BZP (6.0 mg/kg) multiple**	
	**Male**	**Female**	**Male**	**Female**	**Male**	**Female**	**Male**	**Female**
AUC_0–t_ (mg·h/L)	56.09 ± 2.98	71.73 ± 10.83^*^	145.43 ± 22.33	174.18 ± 36.35	222.78 ± 56.04	273.12 ± 39.52	179.54 ± 12.73	151.52 ± 70.08
AUC_0–∞_ (mg·h/L)	56.23 ± 3.04	71.99 ± 10.75^*^	145.67 ± 22.37	174.62 ± 36.48	223.63 ± 55.72	275.07 ± 39.90	179.96 ± 12.76	152.05 ± 69.90
MRT_0–t_ (min)	15.84 ± 3.45	19.24 ± 4.62	14.81 ± 1.94	15.79 ± 1.78	19.75 ± 1.66	22.66 ± 5.08	15.53 ± 2.37	19.18 ± 2.88
MRT_0–∞_ (min)	16.26 ± 3.28	19.81 ± 4.37	15.23 ± 2.29	16.27 ± 1.93	21.52 ± 2.33	26.08 ± 5.59	16.18 ± 2.75	20.01 ± 3.25
t_1/2z_ (min)	22.70 ± 5.80	25.52 ± 9.70^*^	26.78 ± 12.37^Δ^	24.53 ± 3.03^*^^Δ^	64.86 ± 19.59^ΔΔ^	95.59 ± 23.09^*^^ΔΔ^	35.84 ± 10.48	28.74 ± 9.21^*^
CL_z_ (L/min·kg)	0.054 ± 0.003	0.043 ± 0.008^*^	0.042 ± 0.006^Δ^	0.035 ± 0.008^*^^Δ^	0.056 ± 0.012^Δ^	0.045 ± 0.007^*^^Δ^	0.033 ± 0.003	0.046 ± 0.02^*^
V_z_ (L/kg)	1.74 ± 0.37	1.52 ± 0.44	1.63 ± 0.84^ΔΔ^	1.25 ± 0.25^ΔΔ^	5.48 ± 2.57^ΔΔ^	6.06 ± 1.38^ΔΔ^	1.72 ± 0.51	1.78 ± 0.57
*C*_max_	5880 ± 827	6359 ± 946	15,048 ± 3156	15,382 ± 2013	22,030 ± 4007	20,750 ± 5674	16,644 ± 2827	10,438 ± 5736

* P < 0.05, compared with male group;

Δ *P < 0.05*,

ΔΔ P < 0.01, compared with the group (3.0 mg/kg).

##### PK profile of Br-NBP after multiple dose of BZP in rats

As shown in Figure 3, Table 2, the *C*_min_ values of Br-NBP were under the LLOQ (5 ng/mL) at 5–7 day. The *C*_max_ and AUC_0–t_ values of Br-NBP had no significant differences in female and male rats after 7 consecutive day's administration. Compared with the parameters after single dose of BZP (6 mg/kg, *i.v.*), the *C*_max_, AUC_0–t_, CL_z_, and the t_1/2_ values of Br-NBP had no significant differences after 7 consecutive day's administration.

#### *In vivo* PK profile in beagle dogs

##### Single dose of BZP in beagle dogs

**Figure 4:**
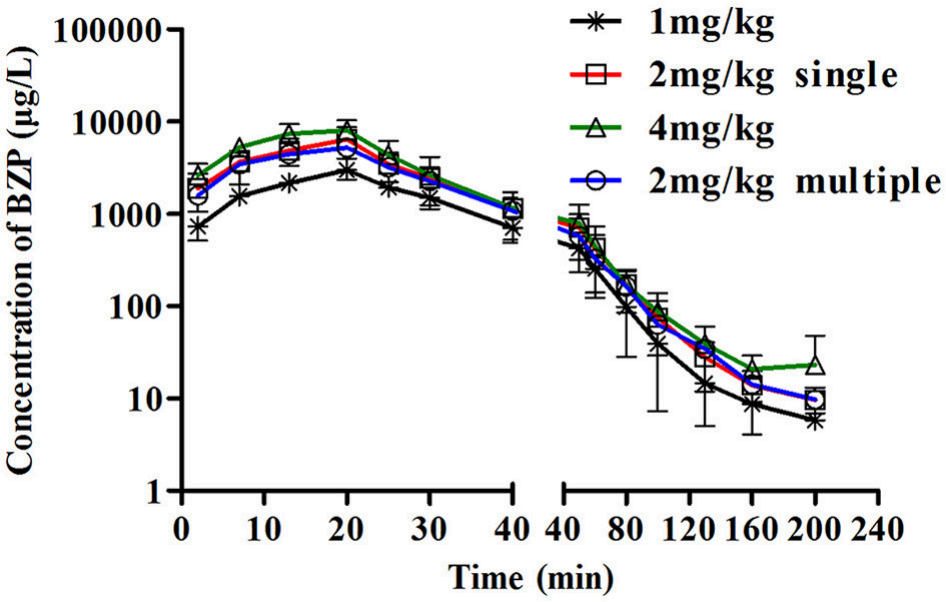
**The mean plasma concentration-time profiles of BZP in beagle dogs**. Beagle dogs received intravenous drip administration of BZP at a single dose of 1, 2 or 4 mg/kg (*n* = 8) and at multiple dose of 2 mg/kg for 7 days (*n* = 8). The results are the mean ± SD of the indicated number of beagle dogs.

The beagle dogs in the test groups were intravenous drip with BZP at 1, 2, or 4 mg/kg followed by analysis of BZP concentration in plasma. The plots of mean plasma concentration of BZP in dogs were depicted in Figure 4. A summary of the pharmacokinetic parameters for BZP in beagle dogs is shown in Table 3. The mean *C*_max_ and AUC_0–t_ values of BZP were linearly related to the dose in the range of 1–4 mg/kg. The coefficient of association was 0.9124 for dose to AUC_0–t_ in beagle dogs (*y* = 43.103x + 55.3). In addition, for three doses, the AUC_0–t_ and *C*_max_ values of BZP had no significant differences in female and male beagle dogs.

**Table 3:** **The pharmacokinetic parameters of BZP after single and multiple intravenous drip administration of BZP in beagle dogs (***n*** = 8)**.

**PK parameters**	**Single**			**Multiple**
	**BZP (1.0 mg/kg)**	**BZP (2.0 mg/kg)**	**BZP (4.0 mg/kg)**	**BZP (2.0 mg/kg)**
AUC_0–t_ (mg·h/L)	82.98 ± 14.14	164.64 ± 49.84	210.61 ± 67.27	146.07 ± 44.49
AUC_0–∞_ (mg·h/L)	83.10 ± 14.14	165.04 ± 49.93	211.10 ± 67.34	146.46 ± 44.58
MRT_0–t_ (min)	26.98 ± 3.53	25.22 ± 1.76	23.47 ± 1.87^*^	25.36 ± 2.97
MRT_0–∞_ (min)	27.25 ± 3.57	25.82 ± 1.61	24.01 ± 1.73^*^^Δ^	25.09 ± 2.93
t_1/2z_ (min)	22.26 ± 9.05	34.62 ± 18.31	33.05 ± 6.98^*^	35.20 ± 11.70
CL_z_ (L/min·kg)	0.012 ± 0.002	0.014 ± 0.008	0.021 ± 0.005^**^	0.015 ± 0.005
V_z_ (L/kg)	0.40 ± 0.20	0.70 ± 0.50	1.01 ± 0.45^**^	0.77 ± 0.39
*C*_max_ (ng/mL)	3008 ± 651	6463 ± 2249	8096 ± 2235	5235 ± 1244

* *P < 0.05*,

** P < 0.01, compared with the group (1.0 mg/kg);

Δ P < 0.05, compared with the single dose group (2.0 mg/kg).

##### Multiple dose of BZP in beagle dogs

As presented in Figure 4, Table 3, the *C*_min_ values of BZP were under the LLOQ (5 ng/mL) at 5–7 day. The *C*_max_ and AUC_0–t_ values of BZP had no significant differences in female and male rats after 7 consecutive day's administration. Compared with the parameters after single dose of BZP (2 mg/kg, *i.v.gtt*), the *C*_max_ and AUC_0–t_, CL_z_, V_z_, and t_1/2_ values had no significant differences after 7 consecutive day's administration.

##### PK profile of Br-NBP after single dose of BZP in beagle dogs

**Figure 5:**
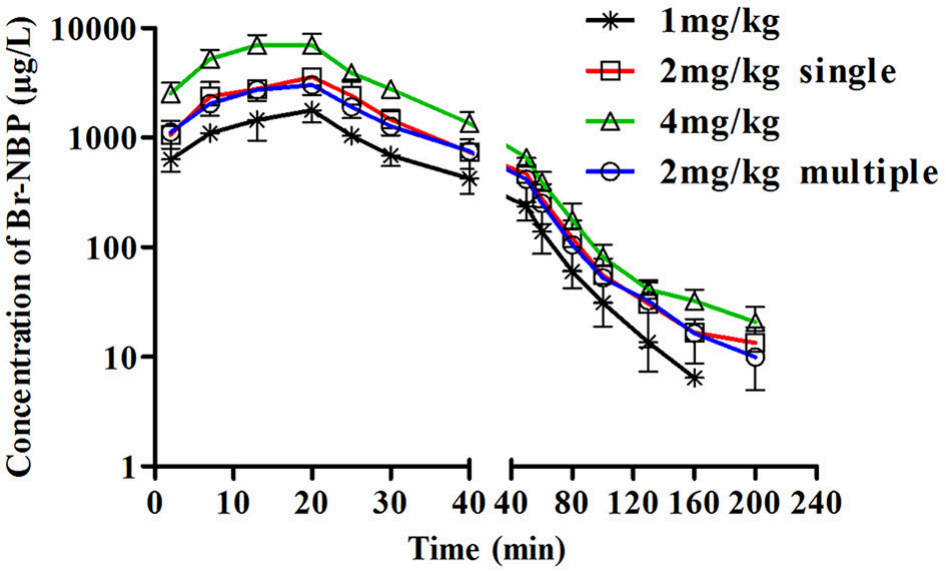
**The mean plasma concentration-time profiles of Br-NBP in beagle dogs**. Beagle dogs received intravenous drip administration of BZP at single dose of 1, 2 or 4 mg/kg (*n* = 8) and at multiple dose of 2 mg/kg for 7 days (*n* = 8). The results are the mean ± SD of the indicated number of beagle dogs.

The mean plasma concentration-time profiles of Br-NBP after administration of BZP (1, 2, and 4 mg/kg, *i.v.gtt*) were illustrated in Figure 5. The pharmacokinetic parameters of Br-NBP were presented in Table 4. The mean *C*_max_ and AUC_0–t_ values of Br-NBP were linearly related to the dose in the range of 1–4 mg/kg. The coefficient of association was 0.9999 for dose to AUC_0–t_ in beagle dogs (*y* = 49.837x + 0.62). In addition, for three doses, the AUC_0–t_ and *C*_max_ values of Br-NBP had no significant differences in female and male beagle dogs. The other major parameters such as t_1/2z_, CL_z_, and V_z_ values exhibited no differences in the range of 1–4 mg/kg.

**Table 4:** **The pharmacokinetic parameters of Br-NBP in beagle dogs after single and multiple intravenous drip administration of BZP (***n*** = 8)**.

**PK Parameters**	**Single**			**Multiple**
	**BZP (1.0 mg/kg)**	**BZP (2.0 mg/kg)**	**BZP (4.0 mg/kg)**	**BZP (2.0 mg/kg)**
AUC_0–t_ (mg·h/L)	49.86 ± 5.97	101.19 ± 17.75	199.67 ± 26.77	91.04 ± 14.48
AUC_0–∞_ (mg·h/L)	50.15 ± 5.98	101.69 ± 17.92	200.91 ± 27.15	91.52 ± 14.34
MRT_0–t_ (min)	26.19 ± 2.87	26.84 ± 2.55	24.51 ± 2.19^Δ^	27.40 ± 2.70
MRT_0–∞_ (min)	27.05 ± 3.39	27.84 ± 2.71	26.10 ± 2.14^Δ^	28.74 ± 3.05
t_1/2z_ (min)	21.57 ± 6.54	31.90 ± 7.62	43.08 ± 22.29^Δ^	36.91 ± 13.29
CL_z_ (L/min·kg)	0.020 ± 0.002	0.020 ± 0.005	0.020 ± 0.003^ΔΔ^	0.022 ± 0.003
V_z_ (L/kg)	0.62 ± 0.14	0.94 ± 0.34	1.24 ± 0.60^ΔΔ^	1.21 ± 0.52
*C*_max_ (ng/mL)	1896 ± 303	3714 ± 677	7436 ± 1790	3101 ± 512

Δ P < 0.05,

ΔΔ P < 0.01, compared with the group (2.0 mg/kg).

##### PK profile of Br-NBP after multiple dose of BZP in beagle dogs

The mean plasma concentration-time profiles of Br-NBP after BZP treatment (2 mg/kg, once daily for 7 days, *i.v.gtt*) were shown in Figure 5. The pharmacokinetic parameters of Br-NBP are presented in Table 4. The *C*_min_ values of Br-NBP are under LLOQ (5 ng/mL) at 5–7 days. The *C*_max_ and AUC_0–t_ values of Br-NBP were no significant differences in female and male rats after 7 consecutive day's administration. Compared with the parameters after single dose of BZP (2 mg/kg, *i.v.gtt*), the *C*_max_, and AUC_0–t_, CL_z_, V_z_, and t_1/2_ values had no significant differences after 7 consecutive day's administration.

### Tissue distribution

#### The tissue distribution in rats

##### The tissue distribution of BZP in rats

**Figure 6:**
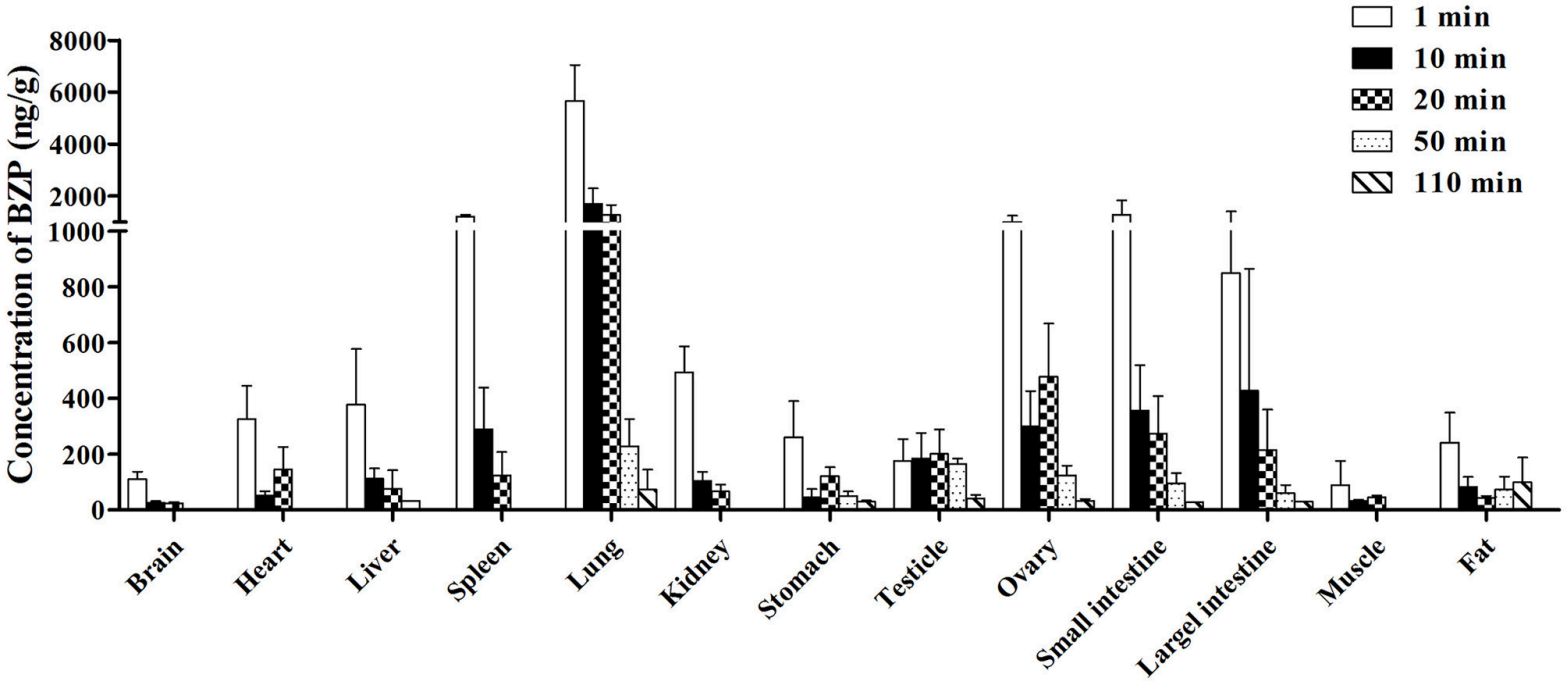
**Mean concentration of BZP in 13 tissues at 1, 10, 20, 50, and 110 min after a single intravenous injection of BZP (6 mg/kg) in rats**. (*n* = 6, mean ± SD).

The concentration of BZP in various tissues of rats determined at 1, 10, 20, 50, and 110 min after *i.v.* administration were presented in Figure 6. At 1 min after *i.v.* injection with a dose of 6 mg/kg, different concentrations of BZP were detected in most tissues. The highest concentration was observed in lung, followed by small intestine, spleen, ovary, large intestine, kidney, liver, heart, stomach, fat, testicle, brain, and muscle. With the prolonging of time, the concentrations of BZP in most of the tissues decreased obviously within 110 min.

##### The tissue distribution of Br-NBP in rats

**Figure 7:**
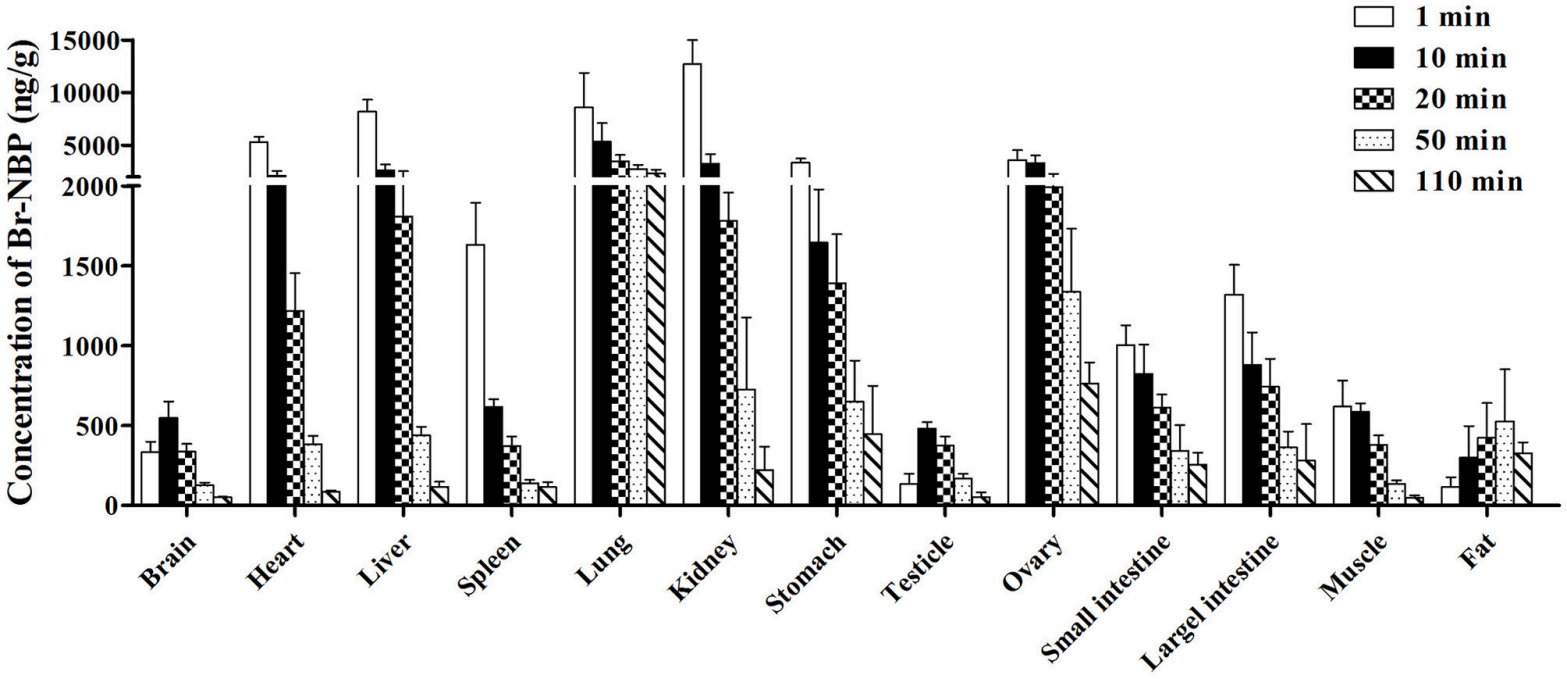
**Mean concentration of Br-NBP in 13 tissues at 1, 10, 20, 50, and 110 min after a single intravenous injection of BZP (6 mg/kg) in rats**. (*n* = 6, mean ± SD).

The concentration of Br-NBP in various tissues of rats determined at 1, 10, 20, 50, and 110 min after administration were presented in Figure 7. The highest concentration was achieved at 1 min in major tissues except for brain and fat. The concentrations of Br-NBP in brain and testicle were elevated from 1 to 10 min and declined till 110 min. At 50 min, the concentration of Br-NBP in fat was highest. Elimination process of Br-NBP from examined tissues was slower compared to BZP. The highest concentration level was observed in kidney, followed by lung, liver, heart, ovary, stomach, spleen, large intestine, small intestine, muscle, brain, and lowest in testicle and fat at 1 min. With the extent of time, the concentrations of Br-NBP in most of the tissues decreased obviously within 110 min.

##### The AUC of BZP and Br-NBP in tissues of rats

The AUC of BZP and Br-NBP in 13 tissues were calculated in trapezoidal method and presented in Table 5. The results indicated that BZP could transform into Br-NBP *in vivo* in major tissues of rats.

**Table 5:** **The AUC_**BZP**_ and AUC_**Br−NBP**_***in vivo*** of rats at 110 min**.

**Tissue**	**AUC_BZP_ (nmol·min/g)**	**AUC_Br−NBP_ (nmol·min/g)**	**AUC_BZP+Br−NBP_ (nmol·min/g)**	**AUCBZP/AUC_Br−NBP_**
Blood^*^	2326.44	504.36	2830.80	4.613
Brain	3.22	77.48	80.71	0.042
Spleen	34.42	113.69	148.11	0.303
Kidney	14.68	607.74	622.43	0.024
Liver	11.91	453.33	465.25	0.026
Heart	10.77	329.40	340.17	0.033
Lung	254.00	1318.30	1572.30	0.193
Ovary	76.09	636.56	712.65	0.120
Testical	49.39	79.91	129.30	0.618
Large intestine	48.60	201.22	249.82	0.242
Small intestine	62.23	177.94	240.17	0.350
Muscle	2.94	88.19	91.13	0.033
Stomach	21.25	377.24	398.49	0.056
Fat	16.30	167.18	183.48	0.098
All	2932.25	5132.55	8064.80	0.571

* For blood: 1 mL ≈ 1.08 g.

#### The distribution of brain in normal and MCAO rats

The concentration of BZP and Br-NBP in the brain at 1 and 10 min after a single intravenous injection of BZP (6 mg/kg) were presented in Table 6. The results indicated that the concentration of BZP had no statistical differences at 1 and 10 min in normal and MCAO rats. But for Br-NBP, the concentration in MCAO rats was much higher than in normal rats at 1 min (*P* < 0.01) and 10 min (*P* < 0.05).

**Table 6:** **The concentration of BZP and Br-NBP at 1 and 10 min after single intravenous injection of BZP (6 mg/kg) in the brain of normal and MCAO rats (ng/g) (***n*** = 6)**.

**Compound**	**Group**	**T(min)**	
		**1**	**10**
BZP	Control	110.69 ± 27.18	26.48 ± 5.16
	MCAO	63.17 ± 18.02	25.66 ± 6.78
Br-NBP	Control	334.35 ± 65.65	547.77 ± 103.85
	MCAO	648.33 ± 53.90^**^	1569.26 ± 421.69^*^

* P < 0.05, compared with control;

** P < 0.01, compared with control.

### Plasma protein binding assay

**Figure 8:**
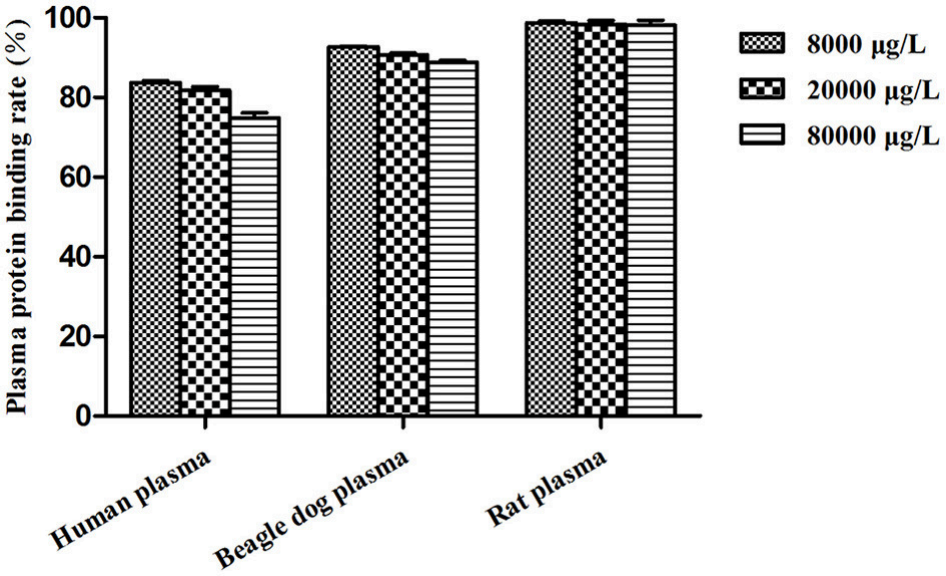
**Plasma protein binding profile of BZP**. (*n* = 3, mean ± SD).

Figure 8 listed the plasma protein binding rates of BZP (%) in pooled plasma samples at different concentrations from pooled SD rat, beagle dog and human plasma. The percent binding was found to be highest in rat plasma with 98.7, 98.4, 98.1% compared in human plasma with 83.7, 81.8, 74.8% and in beagle dog plasma with 92.7, 90.7, 88.9%, respectively.

## Discussion

BZP represents a major breakthrough for an unmet medical need and exerts effective neuroprotective effect on cerebral ischemia-reperfusion damage in rats. The pre-clinical pharmacokinetic study plays an essential role in the evaluation of new drug development. Besides, it is an important basis to evaluate the drugs' characteristics, quality, mechanism of action, basis of efficacy, and toxicity actions. In addition, it can provide vital reference information for the design and optimization of clinical trials (Singh, 2006). As for the pre-clinical pharmacokinetic profiles of BZP were still unclear, we carried out this study to evaluate the pharmacokinetic parameters, tissue distribution and plasma protein binding of BZP and Br-NBP in rodent and non-rodent animals for the first time.

In our study, rodent and non-rodent animals were chosen as the animal models, according to the guidelines of non-clinical pharmacokinetic study approved by CFDA (China Food Drug Administration, 2014). We chose SD rats in rodent animals as the anatomical features and physiological characteristics of SD rats were similar to human. Beagle dogs were selected in non-rodent animals, in consideration of their advanced blood circulation system and similar pharmacology and toxicology reaction with human (Jaiswal et al., 2014). The toxicology studies of BZP were finished in China National Chengdu Center for Safety Evaluation of Drugs under Good laboratory practice of drug (GLP) conditions. The acute toxicity research results showed that, the maximum tolerated doses of BZP were 200 and 300 mg/kg after single *i.v.* administration to rats and beagle dogs, respectively. After single *i.v.* administration to beagle dogs (200, 300 mg/kg), visible tremors, vomiting, weakness, salivation and deep breathing symptoms were shown and could recover in 0.25–4 h. The long-term toxicity results elaborated, the non-toxic-effect dose level of BZP is 50 mg/kg after multiple *i.v.* administration to rats and beagle dogs. General transient toxic symptoms such as head tremor, body tremor, and nervous function toxicity can be seen and recovered in 28 days withdrawal of the drug after multiple *i.v.* administration to rats and beagle dogs. Therefore, the dose levels of BZP selected in this study in rats were generally safe, well tolerated, and in the range of pharmacodynamic effective doses. The conversion of rat doses to beagle dogs equivalent doses based on body surface area was performed in accordance with the guideline set by US FDA [US Department of Health and Human Services (DHHS) et al., 2005].

After intravenous administration of BZP to rats, rapid distribution phase followed by a relatively slow elimination phase of BZP and Br-NBP was observed in concentration-time curves. We have investigated the effect of different doses on the pharmacokinetic behavior of BZP and Br-NBP. The *C*_max_ and AUC_0–t_ of BZP and Br-NBP increased with dose in a dose-dependent manner, which indicated a linear pharmacokinetic profile in 3–12 mg/kg in rats. However, there was a significant difference between the groups for other major pharmacokinetic parameters such as t_1/2z_, CL_z_, and V_z_, which could be caused by the short t_1/2_ level and individual differences. Besides, the AUC_0–t_ ratios of BZP/Br-NBP were 6.08, 3.36, and 4.68 for 3, 6, and 12 mg/kg respectively, suggesting that the dose may influence the transformation of BZP to Br-NBP. After multiple administration of BZP to rats, the trough concentration of this component could not be detected, which demonstrated that there was no accumulation in rats. In addition, the *C*_max_ and AUC_0–t_ of BZP on day 7 were significant higher than on day 1 (*P* < 0.05), which may be due to the enzyme self-inhibition or other factors. The pharmacokinetic parameters and plasma concentrations of Br-NBP between day 1 and day 7 had no difference, which also indicated that there was no accumulation of Br-NBP in rats.

Dose-dependent linear pharmacokinetic profiles of BZP and Br-NBP were observed in beagle dogs after single intravenous drip administration (1, 2, and 4 mg/kg). The volume of distribution was estimated to be more than the total body water, demonstrating extensive distribution to tissues. The short t_1/2_ value contrast to the long t_1/2_ of NBP (*t*_1/2_ = 249 ± 39.0 min) indicated that BZP had fewer side effects (Li et al., 2014). The other pharmacokinetic parameters (t_1/2_, V_z_, and CL) were not agreement in 1–4 mg/kg, which probably because of the short t_1/2_ value and individual differences. Unlike rats, there was no gender difference on the major pharmacokinetic parameters of BZP and Br-NBP in dogs. Furthermore, the AUC_0–t_ ratio of BZP/Br-NBP were 1.66, 1.63, and 1.06 for 1, 2, and 4 mg/kg. No remarkable difference was found among 1–2 mg/kg, but the AUC_0–t_ ratio of BZP/Br-NBP in 4 mg/kg was lower than in 1, 2 mg/kg, suggesting that the dose level has an impact on the transformation of BZP to Br-NBP. After multiple administration of BZP to beagle dogs, the pharmacokinetic parameters and plasma concentrations of BZP and Br-NBP between day 1 and day 7 showed no difference, which indicated that there was no accumulation of BZP and Br-NBP in the plasma of beagle dogs. In a word, the pharmacokinetics of BZP in beagle dogs was quite different from that observed in rats.

Tissue distribution is vital for understanding the major target sites of the drug and provides the information of its disposition *in vivo*, which is closely related to the toxicity and efficacy (Mekjaruskul et al., 2012; Kim et al., 2014). Therefore it is essential to investigate the tissue distribution of BZP and Br-NBP. The sampling points selected for tissue distribution study were in distribution, metabolism and elimination phase. The results demonstrated that BZP and Br-NBP underwent rapid and wide distribution to tissues. The concentration of BZP and Br-NBP in most of the tissues decreased obviously in 110 min, indicating that there was no long-term accumulation, and this concentration variation trend was congruent with that in the plasma. The concentration of BZP in lung, small intestine, and spleen were significantly higher than in other tissues. The highest concentration level of Br-NBP were observed in kidney, lung, liver, heart, which demonstrated that blood flow and perfusion rate of the organ played a key role in the distribution (Shi et al., 2014). The distributions of BZP and Br-NBP were different in major tissues, which could be attributed to the molecules' different solubility proprieties or specific transporters in each tissue (Wagner et al., 2016). In addition, BZP was detectable at a rather low concentration in the brain which illustrated that BZP could transfer across the blood-brain barrier (BBB) and consequently exert significant effect on brain. Additionally, the AUC_0–t_ ratios of BZP/Br-NBP were different in different tissues, which demonstrated the ability and rates of transformation were different. The above results demonstrated that BZP could convert to Br-NBP in rat plasma and tissue through a spontaneous pathway. Br-NBP could be further metabolized into hydroxylation metabolites in liver microsomes by CYP450s (data not shown), while the mechanism of metabolism and excretion still need for further investigation.

It is well accepted that drug's effects is closely related to its target concentration (Billard, 2015). For the purpose of investigating the brain distribution of BZP, we performed further study in MCAO rats, a widely accepted model (Walberer and Rueger, 2015). The results demonstrated the concentrations of BZP were similar in two kinds of rats, there was no significant difference between normal and MCAO rats for the distribution of BZP as its high polar. In addition, the concentration of BZP was largely lower than Br-NBP, which may on account of the high lipid solubility of Br-NBP and spatial structure of halogen atom. Besides, the concentration of Br-NBP in MCAO rats was higher than in normal rats. Experimental and clinical evidence proved that BBB after cerebral ischemia was disrupted and permeability was increased (Ren et al., 2015; Yan et al., 2015). Furthermore, as selective distribution of 10-O*H*-NBP and 3-O*H*-NBP (the hydroxylated metabolites of NBP) across the BBB of rats is mainly attributed to the differences in plasma and brain protein binding and the efflux transport of 3-O*H*-NBP (Diao et al., 2015), the protein binding also could influence the brain distribution.

Plasma protein binding plays a key role in development of pharmacokinetics/pharmacodynamics relationships and prediction of drug–drug interaction potential, potency/selectivity/toxicity, and so on (Riccardi et al., 2015). Pharmacokinetic properties such as hepatic metabolism, distribution volume, and membrane transport are highly related to the unbound fraction of drugs (Tang et al., 2006). Drug-drug interactions may occur by the reason of protein binding displacement, particularly for high protein-binding (>90%) drugs. Such as the anticoagulant drug warfarin, the protein binding rate was about 99%. Lipid-lowering statin drugs fluvastatin (commonly co-administered with warfarin) could significantly displace plasma protein binding of warfarin and induce drug-drug interactions (Shaik et al., 2016). The protein bindings of BZP from various species plasma were different. BZP was more strongly bound to rat and dog plasma than human plasma. However, the plasma binding of BZP in human plasma was not too high, which indicated the drug-drug interactions could not induced by plasma protein binding displacement.

## Conclusion

As a promising agent, the preclinical DMPK data of BZP would be an important asset to exploit its huge therapeutic potential for treatment of ischemic stroke. In present study, the PK profiles of BZP were detailedly assessed in rats and beagle dogs. In conclusion, BZP and Br-NBP showed (A) short half-life, (B) dose linear pharmacokinetic profile, (C) wide tissue distribution, (D) different degree protein binding to various species plasma. The present findings provide valuable information with regards to the experimental PK properties of BZP and Br-NBP in rats and beagle dogs, which serve as a firm basis for further investigation of BZP in both preclinical and clinical phase.

## Author contributions

XT, HL, JW and GW performed the animal experiment, analyzed data and calculated PK parameters, interpreted results of experiments and prepared the manuscript; BL and YZ established the analytical method; HQ and JC designed the whole research and reviewed the final manuscript. All the authors have read and approved the final version.

### Conflict of interest statement

The authors declare that the research was conducted in the absence of any commercial or financial relationships that could be construed as a potential conflict of interest.

## Abbreviations

BZP: sodium (±)-5-bromo-2-(α-hydroxypentyl) benzoate
Br-NBP: 3-butyl-6-bromo-1(3*H*)-isobenzofuranone
LC–MS/MS: liquid chromatography coupled to mass spectrometry
MCAO: middle cerebral artery occlusion
NBP: l-3-n-butylphthalide
CFDA: China Food and Drug Administration
AE: adverse events
PHPB: potassium 2-(1-hydroxypentyl)-benzoate
IS: internal standard
ICA: internal carotid artery
ECA: external carotid artery
MCA: middle cerebral artery
SD: Sprague-Dawley
ESI: electrospray ionization
MRM: multiple reaction monitoring
RSD: relative standard deviation
*AUC*: the area under the plasma concentration–time curve
*C*_max_: the peak plasma concentration
*t*_1/2_: elimination half-life
*CL*_z_: total plasma clearance
*V*_z_: volume of distribution
LLOQ: lower limit of quantification
BBB: blood-brain barrier.
